# Supplementary material for: Transcultural Adaptation of Environmental Health Questionnaire with Attitude, Knowledge, and Skills Scales for Portuguese Nursing Students
Source: Nurs Rep. 2025 Jan 8;15(1):13. doi: 10.3390/nursrep15010013 (PMC11767602; doi:10.3390/nursrep15010013)
Supplement: Supplementary file 1 [file nursrep-15-00013-s001.zip › Supplementary Materials A.pdf]

Supplementary Materials: Children's environmental health questionnaire

English version:

Children's Environmental Health

Sustainability in healthcare means designing and delivering healthcare that uses resources in a way that does not jeopardize future health and well-being.

We ask you to answer ALL the questions, so it's recommended not to spend too much time on any specific question and to answer what you know.

Firstly, demographic data is collected before the questionnaire. Below, you will answer a few questions about your attitudes, knowledge and skills in environmental health.

Your answers will be anonymous and completely confidential.

In advance: THANK YOU FOR YOUR COOPERATION!

Socio-demographic data

Age: ____

Gender: Male Female Transgender

Highest course you are enrolled in: 1st 2nd 3rd 4th

Have you recently (in the last 3 months) attended a session on sustainability and nursing? Yes No Yes, but it's been more than three months

1. Sustainability Attitudes in Nursing Survey

Please indicate the answer you think is most appropriate.

1. **Climate change is an im-portant issue for nursing.**

   Totally disagree 1 2 3 4 5 6 7 Totally agree

2. **Issues about climate change should be included in the nursing curriculum.**

   Totally disagree 1 2 3 4 5 6 7 Totally agree

3. **Sustainability is an im-portant issue for nursing.**

   Totally disagree 1 2 3 4 5 6 7 Totally agree

4. **Sustainability should be included in the nursing curricu-lum.**

   Totally disagree 1 2 3 4 5 6 7 Totally agree

5. **I apply sustainability prin-ciples at home.**

   Totally disagree 1 2 3 4 5 6 7 Totally agree

2. Children´s Environmental Health Knowledge Questionnaire

Please tick the True or False box when considering each statement, or don't know if you don't know the answer.

	Statement	True	False	I don´t know
1.	The paediatric population is more susceptible to environmental threats due to their biological immaturity.	☐	☐	☐
2.	The increased energy and metabolic consumption of the paediatric population protects children from environmental hazards.	☐	☐	☐
3.	The higher rate of cell growth during the paediatric age increases the risk of health effects caused by environmental factors.	☐	☐	☐
4.	Environmental factors do not influence hormonal secretion during puberty.	☐	☐	☐
5.	Nitrogen oxide from fossil fuels in the home and tobacco smoke causes redness and burns on the skin.	☐	☐	☐
6.	Particles from animals exacerbate asthma crisis.	☐	☐	☐
7.	Increased humidity at home improves respiratory diseases in children.	☐	☐	☐
8.	Passive smoking is associated with the development of acute leukemias in children.	☐	☐	☐
9.	Childhood leukemia incidence rates are higher in the areas most exposed to radon.	☐	☐	☐
10.	Overexposure to solar ultraviolet radiations can damage the skin of adults more severely than that of children.	☐	☐	☐
11.	During childhood more than half of the expected lifetime solar ultraviolet radiation is absorbed.	☐	☐	☐
12.	Lead accumulates in the body affecting the nervous system.	☐	☐	☐
13.	Chronic dietary exposure to mercury (fish and shellfish) is less toxic to children's central nervous system than to adults.	☐	☐	☐
14.	Exposure to pesticides increases the risk of developing attention deficit problems in school-aged children.	☐	☐	☐

cc BY NC SA

	True	False	I don´t know
15. Children born to smoking mothers during pregnancy are at risk of lower intellectual capacity.	☐	☐	☐
16. Exposure to organic solvents during fetal development can cause learning disabilities in children.	☐	☐	☐
17. Water containing nitrates can only cause intoxication during childhood.	☐	☐	☐
18. Chlorination of water forms sub-products from the disinfection process that have been classified as carcinogenic.	☐	☐	☐
19. The major source of childhood exposure to pesticides is through ambient air.	☐	☐	☐
20. The main route of exposure to mercury is through cereal intake.	☐	☐	☐
21. Exposure to lead through diet occurs mainly through fish intake.	☐	☐	☐
22. Food colourings and preservatives are associated with central nervous system problems.	☐	☐	☐
23. Genetically modified foods cause fewer allergic reactions in children.	☐	☐	☐
24. Schools and nurseries are environmentally safe places.	☐	☐	☐
25. Children are exposed to higher concentrations of air pollutants at home than outdoors.	☐	☐	☐
26. Parks and gardens are the areas with the least environmental pollutants where children can play.	☐	☐	☐

cc BY NC SA

3. Children's Environmental Health Skills Questionnaire

Please tick the answer from 1 to 5 as you feel each statement corresponds to you.

1. I am able to assess the main environmental risks to which a child is exposed.

   Strongly disagree 1 2 3 4 5 Strongly agree

2. I am NOT able to identify the environmental risks that can cause respiratory diseases in a child.

   Strongly disagree 1 2 3 4 5 Strongly agree

3. I am able to identify the environmental risks that can cause neoplastic diseases in a child.

   Strongly disagree 1 2 3 4 5 Strongly agree

4. I am NOT able to identify the environmental risks that can cause neurological disorders in a child.

   Strongly disagree 1 2 3 4 5 Strongly agree

5. I am able to provide health education to parents about the main contaminants in their child's food.

   Strongly disagree 1 2 3 4 5 Strongly agree

6. I am NOT able to identify the environmental risks in playgrounds.

   Strongly disagree 1 2 3 4 5 Strongly agree

7. I am able to provide health education to parents about actions to minimize environmental risks to which a child is exposed when playing outdoors.

   Strongly disagree 1 2 3 4 5 Strongly agree

8. I am NOT able to identify the environmental risks in a child's home.

   Strongly disagree 1 2 3 4 5 Strongly agree

9. I am able to provide health promotion to parents about environmental risks at home.

   Strongly disagree 1 2 3 4 5 Strongly agree

10. I am able to identify the environmental risks in a child's school.

    Strongly disagree 1 2 3 4 5 Strongly agree

11. I am NOT able to identify the actions needed to combat environmental risks in a child's school.

    Strongly disagree 1 2 3 4 5 Strongly agree

12. I do NOT feel able to do my job as a nurse in a Pediatric Environmental Health Specialty Unit.

    Strongly disagree 1 2 3 4 5 Strongly agree

cc BY NC SA

Original portuguese version:

Saúde Ambiental Infantil

Sustentabilidade nos cuidados de saúde significa conceber e prestar cuidados de saúde que utilizem recursos de forma a não prejudicar a saúde e o bem-estar futuros.

Pedimos-lhe que responda a TODAS as perguntas, pelo que é recomendado não gastar muito tempo com qualquer pergunta específica e responder ao que sabe.

Em primeiro lugar, antes do questionário, são recolhidos dados demográficos. Abaixo, responderá a algumas perguntas sobre as suas atitudes, conhecimentos e competências em matéria de saúde ambiental.

As suas respostas serão anónimas e completamente confidenciais.

Com antecedência: MUITO OBRIGADO PELA SUA COLABORAÇÃO!

Dados sociodemográficos

Idade: ____

Género: ☐Masculino ☐Feminino ☐Transgénero

Curso mais alto em que está inscrito: ☐1º ☐2º ☐3º ☐4º

Assistiu recentemente (nos últimos 3 meses) a uma sessão sobre sustentabilidade e enfermagem? ☐Sim ☐Não ☐Sim, mas já se passaram mais de três meses

1. Pesquisa sobre Atitudes Promotoras de Sustentabilidade em Enfermagem

Por favor, indique a resposta que considera mais apropriada.

1. **O tema das alterações climáticas é importante para a enfermagem.**

   Totalmente em desacordo 1 2 3 4 5 6 7 Totalmente de acordo

2. **Os temas sobre alterações climáticas devem ser incluídos no plano currícular de enfermagem.**

   Totalmente em desacordo 1 2 3 4 5 6 7 Totalmente de acordo

3. **A sustentabilidade é um tema importante para a enfermagem.**

   Totalmente em desacordo 1 2 3 4 5 6 7 Totalmente de acordo

4. **A sustentabilidade deve ser incluída no plano curricular de enfermagem.**

   Totalmente em desacordo 1 2 3 4 5 6 7 Totalmente de acordo

5. **Aplico os principios da sustentabilidade em casa.**

   Totalmente em desacordo 1 2 3 4 5 6 7 Totalmente de acordo

2. Questionário de Habilidades de Saúde Ambiental Infantil

Por favor marque a caixa Verdadeiro ou Falso ao considerar cada declaração, ou não sei se não sabe a resposta.

	Verdadeiro	Falso	Não sei
**27.** A população pediátrica é mais suscetível às ameaças ambientais devido à sua imaturidade biológica.	☐	☐	☐
**28.** O maior consumo energético e metabólico da população pediátrica protege as crianças dos riscos ambientais.	☐	☐	☐
**29.** A maior taxa de crescimento celular na idade pediátrica aumenta o risco de efeitos na saúde decorrentes de fatores ambientais.	☐	☐	☐
**30.** Os fatores ambientais não influenciam a secreção hormonal durante a puberdade.	☐	☐	☐
**31.** O oxido de nitrogénio procedente de combustiveis fosseis provenientes de habitos domesticos e fumo de tabaco produzem vermelhão e queimaduras na pele.	☐	☐	☐
**32.** Partículas provenientes de animais agravam ataques de asma.	☐	☐	☐
**33.** O aumento da humidade ambiente em casa melhora as doenças respiratórias nas crianças.	☐	☐	☐
**34.** O tabagismo passivo está associado ao desenvolvimento de leucemia aguda na infancia.	☐	☐	☐
**35.** As taxas de incidência de leucemia infantil são maiores em áreas com maior exposição a radão.	☐	☐	☐
**36.** A sobreexposição à radiação ultravioleta solar pode danificar a pele de adultos mais intensamente do que a das crianças.	☐	☐	☐
**37.** Durante a infancia é absorbida mais de metade da radiação ultravioleta solar esperada ao longo de toda a vida.	☐	☐	☐
**38.** O chumbo acumula-se no organismo e afeta o sistema nervoso.	☐	☐	☐
**39.** A exposição crónica de mercúrio através da dieta (peixes e mariscos) é menos tóxica para o sistema nervoso central das crianças do que para os adultos.	☐	☐	☐

	Verdadeiro	Falso	Não sei
**40.** **A exposição a agrotóxicos aumenta o risco de desenvolver problemas de déficit de atenção em crianças em idade escolar.**	☐	☐	☐
**41.** **Os filhos de mães que fumam durante a gravidez correm o risco de apresentar menor capacidade intelectual.**	☐	☐	☐
**42.** **A exposição a solventes orgânicos durante o desenvolvimento fetal pode provocar trastornos de aprendizagem na criança.**	☐	☐	☐
**43.** **A água com nitratos pode provocar intoxicação apenas durante a infancia.**	☐	☐	☐
**44.** **A cloração da água forma subprodutos do processo de desinfecção que foram classificados como cancerígenos.**	☐	☐	☐
**45.** **La mayor fuente de exposición infantil a pesticidas es a través del aire ambiental.**	☐	☐	☐
**46.** **A principal via de exposição ao mercúrio é através da ingestão de cereais.**	☐	☐	☐
**47.** **A exposição ao chumbo através da dieta decorre principalmente da ingestão de peixe.**	☐	☐	☐
**48.** **Os Corantes e conservantes alimentares estão associados a alterações no sistema nervoso central.**	☐	☐	☐
**49.** **Os Alimentos geneticamente modificados produzem menos reações alérgicas em crianças.**	☐	☐	☐
**50.** **Las escuelas y guarderías son lugares libres de riesgos medioambientales.**	☐	☐	☐
**51.** **Los niños y niñas están expuestos a mayores concentraciones de contaminantes atmosféricos en casa que en el exterior.**	☐	☐	☐
**52.** **Os parques e jardins são as áreas mais livres de contaminantes ambientais em que as crianças podem brincar.**	☐	☐	☐

3. Questionário de Habilidades de Saúde Ambiental Infantil

Por favor, assinale a resposta de 1 a 5, pois sente que cada declaração lhe corresponde.

13. **Sou capaz de avaliar os principais riscos ambientais a que uma criança está exposta.**

Totalmente em desacordo 1 2 3 4 5 Totalmente de acordo

14. **NÃO sou capaz de identificar os riscos ambientais que podem causar doenças respiratórias numa criança.**

Totalmente em desacordo 1 2 3 4 5 Totalmente de acordo

15. **Sou capaz de reconhecer os riscos ambientais que podem causar doenças neoplásicas numa criança.**

Totalmente em desacordo 1 2 3 4 5 Totalmente de acordo

16. **NÃO sou capaz de identificar os riscos ambientais que podem causar doenças neurológicas numa criança.**

Totalmente em desacordo 1 2 3 4 5 Totalmente de acordo

17. **Sou capaz de realizar educação para a saúde aos pais sobre os principais contaminantes presentes na alimentação dos seus filhos.**

Totalmente em desacordo 1 2 3 4 5 Totalmente de acordo

18. **NÃO sou capaz de identificar os riscos ambientais existentes nos parques infantis.**

Totalmente em desacordo 1 2 3 4 5 Totalmente de acordo

19. **Sou capaz de realizar educação para a saúde aos pais sobre ações para minimizar os riscos ambientais que uma criança está exposta ao brincar ao ar livre.**

Totalmente em desacordo 1 2 3 4 5 Totalmente de acordo

20. **NÃO consigo identificar os riscos ambientais existentes em casa de uma criança.**

Totalmente em desacordo 1 2 3 4 5 Totalmente de acordo

21. **Sou capaz de realizar educação para a saúde aos pais sobre os riscos ambientais existentes em casa.**

Totalmente em desacordo 1 2 3 4 5 Totalmente de acordo

22. **Sou capaz de identificar os riscos ambientais existentes na escola de uma criança.**

Totalmente em desacordo 1 2 3 4 5 Totalmente de acordo

23. **NÃO consigo identificar as ações necessárias para combater os riscos ambientais existentes na escola de uma criança.**

Totalmente em desacordo 1 2 3 4 5 Totalmente de acordo

24. **NÃO me sinto capaz de realizar o meu trabalho como enfermeiro numa Unidade de Saúde Ambiental Pediátrica – Unidade de Saúde Pública na área da Saúde Ambiental e Saúde Escolar.**

Totalmente em desacordo 1 2 3 4 5 Totalmente de acordo
