# Supplementary material for: Transcultural Adaptation of Environmental Health Questionnaire with Attitude, Knowledge, and Skills Scales for Portuguese Nursing Students
Source: Nurs Rep. 2025 Jan 8;15(1):13. doi: 10.3390/nursrep15010013 (PMC11767602; doi:10.3390/nursrep15010013)
Supplement: Supplementary file 1 [file nursrep-15-00013-s001.zip › Supplementary Materials B.pdf]

Supplementary Materials B: Translation and back-translation master data

SANS_2

Encuesta de Actitudes en sostenibilidad para Enfermería

Pesquisa sobre Atitudes Promotoras de Sustentabilidade em Enfermagem

1. El cambio climático es un tema importante para enfermería

Traducción 1: O tema das alterações climáticas é importante para a enfermagem.
Traducción 2: O tema das alterações climáticas é importante para a enfermagem.
Retrotraducción 1: El tema de las alteraciones climáticas es importante para enfermería.
Retrotraducción 2: Las alteraciones climáticas son importantes para enfermería.

Item final: O tema das alterações climáticas é importante para a enfermagem.

2. Los temas sobre cambio climático deben ser incluidos en el currículum de enfermería

Traducción 1: Os temas sobre alterações climáticas devem ser incluídos no plano currícular de enfermagem.
Traducción 2: Os tópicos sobre alterações climáticas devem ser incluídos no plano currícular de enfermagem.
Retrotraducción 1: Los temas sobre cambio climático deben ser incluidos en el curriculum de enfermería
Retrotraducción 2: Contenidos sobre alteraciones climáticas deben ser incluidos en el currículo de Enfermería.

Ítem final: Os temas sobre alterações climáticas devem ser incluídos no plano currícular de enfermagem.

3. La sostenibilidad es un tema importante para enfermería

Traducción 1: A sustentabilidade é um tema importante para a enfermagem.
Traducción 2: A sustentabilidade é uma área importante para a enfermagem.
Retrotraducción 1: La sostenibilidad es un tema importante para enfermería.
Retrotraducción 2: La sostenibilidad es un área importante para enfermería.

Ítem final: A sustentabilidade é um tema importante para a enfermagem.

4. La sostenibilidad debe ser incluida en el currículum de enfermería

Traducción 1: A sustentabilidade deve ser incluída no plano curricular de enfermagem
Traducción 2: A sustentabilidade debe ser incluída no plano curricular de enfermagem

Retrotraducción 1: La sostenibilidad debe ser incluida en el curriculum de enfermería.<br>Retrotraducción 2: La sostenibilidad debe ser incluida en el currículo de Enfermería.<br><br>**Item final:** A sustentabilidade deve ser incluída no plano curricular de enfermagem.
**5. Aplico los principios de sostenibilidad en el hogar**<br><br>Traducción 1: Aplico os principios da sustentabilidade em casa.<br>Traducción 2: Aplico os princípios da sustentabilidade em casa.<br>Retrotraducción 1: Aplico los principios de sostenibilidad en casa.<br>Retrotraducción 2: Aplico los principios de sostenibilidad en casa.<br><br>**Ítem final:** Aplico os principios da sustentabilidade em casa.

Respuestas:

Totalmente en desacuerdo Totalmente de acuerdo

1 2 3 4 5 6 7

Traducción:

Totalmente em desacordo **Totalmente de acordo**

1 2 3 4 5 6 7

COMENTARIOS:

ChEHK-Q

Cuestionario de Habilidades en Salud Medioambiental Infantil

Questionário de Habilidades de Saúde Ambiental Infantil

1. La población pediátrica es más susceptible a las amenazas medioambientales debido a su inmadurez biológica.

Traducción 1: A população pediátrica é mais suscetível às ameaças ambientais devido à sua imaturidade biológica.
Traducción 2: A população pediátrica é mais suscetível às ameaças ambientais devido à sua imaturidade biológica.
Retrotraducción 1: La población pediátrica es más susceptible a las amenazas ambientales debido a su inmadurez biológica.
Retrotraducción 2: La población pediátrica es más susceptible de amenazas ambientales debido a su inmadurez biológica.

Ítem final: A população pediátrica é mais suscetível às ameaças ambientais devido à sua imaturidade biológica.

2. El mayor consumo energético y metabólico de la población pediátrica protege a la infancia de los peligros medioambientales.

Traducción 1: O maior consumo energético e metabólico da população pediátrica protege as crianças dos riscos ambientais.
Traducción 2: O maior consumo energético e metabólico da população pediátrica protege as crianças dos riscos ambientais.
Retrotraducción 1: El mayor consumo energético y metabólico de la población pediátrica protege a la infancia de los peligros medioambientales.
Retrotraducción 2: El mayor consumo energético y metabólico de la población pediátrica protege a los niños de los riesgos ambientales.

Ítem final: O maior consumo energético e metabólico da população pediátrica protege as crianças dos riscos ambientais.

3. La mayor tasa de crecimiento celular en la edad pediátrica incrementa el riesgo de efectos para la salud derivados de factores medioambientales.

Traducción 1: A maior taxa de crescimento celular na idade pediátrica aumenta o risco de efeitos na saúde decorrentes de fatores ambientais
Traducción 2: A maior taxa de crescimento celular na idade pediátrica aumenta o risco de efeitos na saúde decorrentes de fatores ambientais
Retrotraducción 1: La mayor tasa de crecimiento celular en la edad pediátrica aumenta el riesgo de efectos en la salud derivados de factores mediambientales.
Retrotraducción 2: La mayor tasa de crecimiento celular en la edad pediátrica aumenta el riesgo de efectos en la salud que surgen de factores ambientales.

Ítem final: A maior taxa de crescimento celular na idade pediátrica aumenta o risco de efeitos na saúde decorrentes de fatores ambientais.

4. Los factores medioambientales no influyen en la secreción hormonal durante la pubertad.

Traducción 1: Os fatores ambientais não influenciam a secreção hormonal durante a puberdade.
Traducción 2: Os fatores ambientais não influenciam a secreção hormonal durante a puberdade.
Retrotraducción 1: Los factores ambientales no influyen en la secreción hormonal durante la pubertad.
Retrotraducción 2: Los factores ambientales no influencian la secreción hormonal durante la pubertad.

Ítem final: Os fatores ambientais não influenciam a secreção hormonal durante a puberdade.

5. El óxido de nitrógeno procedente de combustibles fósiles en el hogar y humo de tabaco producen enrojecimiento y quemaduras en la piel.

Traducción 1: O oxido de nitrogénio procedente de combustiveis fosseis provenientes de habitos domesticos e fumo de tabaco produzem vermelhão e queimaduras na pele.
Traducción 2: O oxido de nitrogénio procedente de combustiveis fosseis provenientes de consumos domesticos e do fumo de tabaco produzem eritema e queimaduras cutáneas.
Retrotraducción 1: El óxido de nitrógeno procedente de combustibles fósiles procedentes de hábitos domésticos y humo de tabaco producen enrojecimiento y quemaduras en la piel.
Retrotraducción 2: El óxido de nitrógeno proveniente de los combustibles fósiles de hábitos domésticos y el humo del tabaco producen enrojecimiento y quemaduras en la piel.

Ítem final: O oxido de nitrogénio procedente de combustiveis fosseis provenientes de habitos domesticos e fumo de tabaco produzem vermelhão e queimaduras na pele.

6. Las partículas procedentes de animales agravan las crisis asmáticas.

Traducción 1: Partículas provenientes de animais agravam ataques de asma.
Traducción 2: Partículas provenientes de animais agravam ataques de asma.
Retrotraducción 1: Las partículas procedentes de animales agravan los ataques de asma.
Retrotraducción 2: Las partículas provenientes de animales empeoran los ataques de asma.

Ítem final: Partículas provenientes de animais agravam ataques de asma.

7. El aumento de la humedad ambiental del hogar mejora las enfermedades respiratorias en los niños y las niñas.

Traducción 1: O aumento da humidade ambiente em casa melhora as doenças respiratórias nas crianças.

Traducción 2: O aumento da humidade ambiente em casa melhora as doenças respiratórias nas crianças.
Retrotraducción 1: El aumento de la humedad ambiental en el hogar mejora las enfermedades respiratorias en los niños.
Retrotraducción 2: El aumento de la humedad en el ambiente en casa mejora las patologías respiratorias en los niños.

Ítem final: O aumento da humidade ambiente em casa melhora as doenças respiratórias nas crianças.

8. El tabaquismo pasivo está asociado al desarrollo de leucemias agudas en la infancia.

Traducción 1: O tabagismo passivo está associado ao desenvolvimento de leucemia aguda na infancia.
Traducción 2: O tabagismo passivo está associado ao desenvolvimento de leucemia aguda na infancia.
Retrotraducción 1: El tabaquismo pasivo está asociado al desarrollo de leucemias aguda en la infancia.
Retrotraducción 2: El tabaquismo pasivo está asociado al desarrollo de leucemia aguda en la infancia.

Ítem final: O tabagismo passivo está associado ao desenvolvimento de leucemia aguda na infancia.

9. Las tasas de incidencia de leucemia infantil son mayores en las zonas con mayor exposición a radón.

Traducción 1: As taxas de incidência de leucemia infantil são maiores em áreas com maior exposição a radão.
Traducción 2: As taxas de incidência de leucemia infantil são maiores em áreas com maior exposição a radão.
Retrotraducción 1: Las tasas de incidencia de leucemia infantil son mayores en las zonas con mayor exposición a radiación.
Retrotraducción 2: Las tasas de incidencia de leucemia infantil son mayores en áreas con mayor exposición a la radiación.

Ítem final: As taxas de incidência de leucemia infantil são maiores em áreas com maior exposição a radão.

10. La sobreexposición a las radiaciones ultravioletas solares puede dañar más intensamente la piel de los adultos que la de los niños y las niñas.

Traducción 1: A sobreexposição à radiação ultravioleta solar pode danificar a pele de adultos mais intensamente do que a das crianças.
Traducción 2: A sobreexposição à radiação ultravioleta solar pode danificar a pele de adultos mais intensamente do que a das crianças.
Retrotraducción 1: La sobreexposición a las radiaciones ultravioleta solares puede dañar la piel de los adultos más intensamente que la de los niños.

Retrotraducción 2: La sobreexposición a la radiación UV solar puede dañar la piel de los adultos más que la de los niños.<br><br>Ítem final: A sobreexposição à radiação ultravioleta solar pode danificar a pele de adultos mais intensamente do que a das crianças.
**11.** Durante la infancia se absorbe más de la mitad de la radiación ultravioleta solar esperada a lo largo de toda la vida.<br><br>Traducción 1: Durante a infancia é absorbida mais de metade da radiação ultravioleta solar esperada ao longo de toda a vida.<br>Traducción 2: Durante a infancia é absorbida mais de metade da radiação ultravioleta solar esperada ao longo de toda a vida.<br>Retrotraducción 1: Durante la infancia se absorbe más de la mitad de la radiación ultravioleta solar esperada a lo largo de toda la vida.<br>Retrotraducción 2: Durante la infancia se absorbe más de la mitad de radiación UV solar esperada para toda la vida.<br><br>**Ítem final:** Durante a infancia é absorbida mais de metade da radiação ultravioleta solar esperada ao longo de toda a vida.
**12.** El plomo se acumula en el organismo afectando al sistema nervioso.<br><br>Traducción 1: O chumbo acumula-se no organismo e afeta o sistema nervoso.<br>Traducción 2: O chumbo acumula-se no organismo e afeta o sistema nervoso.<br>Retrotraducción 1: El plomo se acumula en el organismo y afecta al sistema nervioso.<br>Retrotraducción 2: El plomo se acumula en el organismo y afecta al sistema nervioso.<br><br>**Ítem final:** O chumbo acumula-se no organismo e afeta o sistema nervoso.
**13.** La exposición crónica al mercurio a través de la dieta (pescados y mariscos) es menos tóxica para el sistema nervioso central de los niños y las niñas que para el de los adultos.<br><br>Traducción 1: A exposição crónica de mercúrio através da dieta (peixes e mariscos) é menos tóxica para o sistema nervoso central das crianças do que para os adultos.<br>Traducción 2: A exposição crónica de mercúrio através da dieta (peixes e mariscos) é menos tóxica para o sistema nervoso central das crianças do que para os adultos.<br>Retrotraducción 1: La exposición crónica al mercurio a través de la dieta (pescados y mariscos) es menos tóxica para el sistema nervioso central de los niños que para el de los adultos.<br>Retrotraducción 2: La exposición crónica de mercurio a través de la dieta (pescados y mariscos) es menos tóxica para el sistema nervioso central de los niños que de los adultos.<br><br>**Ítem final:** A exposição crónica de mercúrio através da dieta (peixes e mariscos) é menos tóxica para o sistema nervoso central das crianças do que para os adultos.

14. La exposición a plaguicidas aumenta el riesgo de desarrollar problemas de déficit de atención en escolares.

Traducción 1: A exposição a agrotóxicos aumenta o risco de desenvolver problemas de déficit de atenção em crianças em idade escolar.
Traducción 2: A exposição a agrotóxicos aumenta o risco de desenvolver problemas de déficit de atenção em crianças em idade escolar
Retrotraducción 1: La exposición a plaguicidas aumenta el riesgo de desarrollar problemas de déficit de atención en niños en los escolares.
Retrotraducción 2: La exposición a pesticidas aumenta el riesgo de desarrollar problemas de déficit de atención en niños en edad escolar.

Ítem final: A exposição a agrotóxicos aumenta o risco de desenvolver problemas de déficit de atenção em crianças em idade escolar.

15. Los hijos e hijas de madres fumadoras durante el embarazo tienen riesgo de presentar menor capacidad intelectual.

Traducción 1: Os filhos de mães que fumam durante a gravidez correm o risco de apresentar menor capacidade intelectual.
Traducción 2: Os filhos de mães que fumam durante a gravidez correm o risco de apresentar menor capacidade intelectual.
Retrotraducción 1: Los hijos de madres fumadoras durante el embarazo tienen riesgo de presentar menor capacidad intelectual.
Retrotraducción 2: Los hijos de madres que fuman durante el embarazo corren el riesgo de presentar una menor capacidad intelectual.

Ítem final: Os filhos de mães que fumam durante a gravidez correm o risco de apresentar menor capacidade intelectual.

16. La exposición a solventes orgánicos durante el desarrollo fetal puede provocar trastornos del aprendizaje en un niño o una niña.

Traducción 1: A exposição a solventes orgânicos durante o desenvolvimento fetal pode provocar trastornos de aprendizagem na criança.
Traducción 2: A exposição a solventes orgânicos durante o desenvolvimento fetal pode provocar dificuldades de aprendizagem na criança.
Retrotraducción 1: La exposición a solventes orgánicos durante el desarrollo fetal puede provocar trastornos del aprendizaje en niños.
Retrotraducción 2: La exposición a solventes orgánicos durante el desarrollo fetal puede provocar trastornos del aprendizaje en niños.

Ítem final: A exposição a solventes orgânicos durante o desenvolvimento fetal pode provocar trastornos de aprendizagem na criança.

17. El agua que contiene nitratos puede producir intoxicación sólo durante la infancia.

Traducción 1: A água com nitratos pode provocar intoxicação apenas durante a infancia.

Traducción 2: Água com nitratos pode provocar intoxicação apenas durante a infancia.
Retrotraducción 1: El agua que contiene nitratos puede producir intoxicación sólo durante la infancia.
Retrotraducción 2: El agua que contiene nitratos puede producir intoxicación sólo durante la infancia.

Ítem final: A água com nitratos pode provocar intoxicação apenas durante a infancia.

18. La cloración del agua forma subproductos del proceso de desinfección que han sido clasificados como cancerígenos.

Traducción 1: A cloração da água forma subprodutos do processo de desinfecção que foram classificados como cancerígenos.
Traducción 2: A cloração (adição de cloro) da água forma subprodutos do processo de desinfecção que foram classificados como cancerígenos.
Retrotraducción 1: La cloración del agua forma subproductos del proceso de desinfección que han sido clasificados como cancerígenos.
Retrotraducción 2: La cloración del agua (añadir cloro) forma subproductos del proceso de desinfección clasificados como cancerígenos.

Ítem final: A cloração da água forma subprodutos do processo de desinfecção que foram classificados como cancerígenos.

19. La mayor fuente de exposición infantil a pesticidas es a través del aire ambiental.

Traducción 1: A maior fonte de exposição infantil a pesticidas é o ar ambiente.
Traducción 2: A maior fonte de exposição infantil a pesticidas é o ar ambiente.
Retrotraducción 1: La mayor fuente de exposición a los plaguicidas en los niños es el aire ambiental.
Retrotraducción 2: La mayor fuente de exposición infantil a pesticidas es a través del aire ambiental.

Ítem final: A maior fonte de exposição infantil a pesticidas é o ar ambiente.

20. La principal vía de exposición al mercurio es a través de la ingesta de cereales.

Traducción 1: A principal via de exposição ao mercúrio é através da ingestão de cereais.
Traducción 2: A principal via de exposição ao mercúrio é a ingestão de cereais.
Retrotraducción 1: La principal vía de exposición al mercurio es a través de la ingesta de cereales.
Retrotraducción 2: La principal vía de exposición al mercurio es a través de la ingesta de cereales.

Ítem final: A principal via de exposição ao mercúrio é através da ingestão de cereais.

21. La exposición al plomo a través de la dieta ocurre principalmente con la ingesta de pescado.

Traducción 1: A exposição ao chumbo através da dieta decorre principalmente da ingestão de peixe.
Traducción 2: A exposição ao chumbo através da dieta decorre principalmente da ingestão de peixe.
Retrotraducción 1: La exposición al plomo a través de la dieta ocurre principalmente con la ingesta de pescado
Retrotraducción 2: La exposición al plomo a través de la dieta ocurre principalmente con la ingesta de pescado.

Ítem final: A exposição ao chumbo através da dieta decorre principalmente da ingestão de peixe.

22. Los colorantes y los conservantes alimentarios se asocian a problemas del sistema nervioso central.

Traducción 1: Os Corantes e conservantes alimentares estão associados a alterações no sistema nervoso central.
Traducción 2: Corantes e conservantes alimentares estão associados a alterações no sistema nervoso central.
Retrotraducción 1: Los colorantes y los conservantes alimentarios se asocian a alteraciones del sistema nervioso central.
Retrotraducción 2: Los colorantes y los conservantes alimentarios se asocian a problemas del sistema nervioso central.

Ítem final: Os Corantes e conservantes alimentares estão associados a alterações no sistema nervoso central.

23. Los alimentos modificados genéticamente producen menos reacciones alérgicas en niños o niñas.

Traducción 1: Os Alimentos geneticamente modificados produzem menos reações alérgicas em crianças.
Traducción 2: Alimentos geneticamente modificados produzem menos reações alérgicas em crianças.
Retrotraducción 1: Los alimentos modificados genéticamente producen menos reacciones alérgicas en niños.
Retrotraducción 2: Los alimentos modificados genéticamente producen menos reacciones alérgicas en niños.

Ítem final: Os Alimentos geneticamente modificados produzem menos reações alérgicas em crianças.

24. Las escuelas y guarderías son lugares libres de riesgos medioambientales.

Traducción 1: As escolas e creches são locais livres de riscos ambientais.
Traducción 2: Escolas e creches são locais livres de riscos ambientais.
Retrotraducción 1: Las escuelas y guarderías son lugares libres de riesgos medioambientales.

Retrotraducción 2: Las escuelas y guarderías son lugares libres de riesgos ambientales.

Ítem final: As escolas e creches são locais livres de riscos ambientais.

25. Los niños y niñas están expuestos a mayores concentraciones de contaminantes atmosféricos en casa que en el exterior.

Traducción 1: As crianças estão expostas a maiores concentrações de poluentes do ar dentro de casa do que no exterior.
Traducción 2: As criançlas estão expostas a maiores concentrações de poluentes do ar dentro de casa do que fora.
Retrotraducción 1: Los niños están expuestos a mayores concentraciones de contaminantes atmosféricos en casa que en el exterior.
Retrotraducción 2: Los niños están expuestos a mayores concentraciones de contaminantes atmosféricos en casa que en el exterior.

Ítem final: As crianças estão expostas a maiores concentrações de poluentes do ar dentro de casa do que no exterior.

26. Los parques y los jardines son las áreas más libres de contaminantes ambientales en las que pueden jugar niños y niñas.

Traducción 1: Os parques e jardins são as áreas mais livres de contaminantes ambientais em que as crianças podem brincar.
Traducción 2: Os parques e jardins são as áreas mais livres de contaminantes ambientais em que as crianças podem brincar.
Retrotraducción 1: Los parques y los jardines son las áreas más libres de contaminantes ambientales en las que pueden jugar los niños.
Retrotraducción 2: Los parques y los jardines son las áreas más libres de contaminantes ambientales en las que pueden jugar los niños.

Ítem final: Os parques e jardins são as áreas mais livres de contaminantes ambientais em que as crianças podem brincar.

Respuestas: Verdadero, Falso, No sé

TRADUCCIÓN: Verdadeiro/falso/Não sei

COMENTARIOS:

ChEHS-Q

Cuestionario de Habilidades en Salud Medioambiental Infantil

Questionário de Habilidades de Saúde Ambiental Infantil

1. Soy capaz de valorar los principales riesgos medioambientales a los que está expuesto un niño o una niña.<br><br>Traducción 1: Sou capaz de avaliar os principais riscos do meio ambiente a que uma criança está exposta.<br>Traducción 2: Sou capaz de avaliar os principais riscos ambientais a que uma criança está exposta.<br>Retrotraducción 1: Soy capaz de valorar los principales riesgos medio ambientales a los que está expuesto un niño.<br>Retrotraducción 2: Soy capaz de valorar los principales riesgos ambientales a los que está expuesto un niño.<br><br>**Ítem final:** Sou capaz de avaliar os principais riscos ambientais a que uma criança está exposta.
2. NO soy capaz de identificar los riesgos medioambientales que pueden producir enfermedades respiratorias en un niño o una niña.<br><br>Traducción 1: Não sou capaz de identificar os riscos do meio ambiente que podem causar doenças respiratórias numa criança.<br>Traducción 2: NÃO sou capaz de identificar os riscos ambientais que podem causar doenças respiratórias numa criança.<br>Retrotraducción 1: NO soy capaz de identificar los riesgos medio ambientales que pueden producir enfermedades respiratorias en un niño<br>Retrotraducción 2: NO soy capaz de identificar los riesgos ambientales que pueden producir enfermedades respiratorias en un niño.<br><br>**Ítem final:** NÃO sou capaz de identificar os riscos ambientais que podem causar doenças respiratórias numa criança.
3. Soy capaz de reconocer los riesgos medioambientales que pueden producir enfermedades neoplásicas en un niño o una niña.<br><br>Traducción 1: Sou capaz de reconhecer os riscos do meio ambiente que podem causar doenças neoplásicas numa criança.<br>Traducción 2: Sou capaz de reconhecer os riscos ambientais que podem causar doenças neoplásicas numa criança.<br>Retrotraducción 1: Soy capaz de reconocer los riesgos medioambientales que pueden producir enfermedades neoplásicas en un niño.<br>Retrotraducción 2: Soy capaz de reconocer los riesgos ambientales que pueden producir enfermedades neoplásicas en un niño.<br><br>**Ítem final:** Sou capaz de reconhecer os riscos ambientais que podem causar doenças neoplásicas numa criança.

**4.** NO soy capaz de identificar los riesgos medioambientales que pueden producir enfermedades neurológicas en un niño o una niña.<br><br>Traducción 1: Não sou capaz de identificar os riscos do meio ambiente que podem causar doenças neurológicas numa criança.<br>Traducción 2: NÃO sou capaz de identificar os riscos ambientais que podem causar doenças neurológicas numa criança.<br>Retrotraducción 1: NO soy capaz de identificar los riesgos medioambientales que pueden producir enfermedades neurológicas en un niño.<br>Retrotraducción 2: NO soy capaz de identificar los riesgos ambientales que pueden producir enfermedades neurológicas en un niño.<br><br>**Ítem final:** NÃO sou capaz de identificar os riscos ambientais que podem causar doenças neurológicas numa criança.
**5.** Soy capaz de realizar educación sanitaria a padres y madres sobre los principales contaminantes presentes en los alimentos de su hijo o hija.<br><br>Traducción 1: Sou capaz de realizar educação para a saúde aos pais sobre os principais contaminantes presentes na alimentação dos seus filhos.<br>Traducción 2: Sou capaz de fazer educação para a saúde aos pais sobre os principais contaminantes presentes na alimentação dos filhos.<br>Retrotraducción 1: Soy capaz de realizar educación sanitaria a padres/ madres sobre los principales contaminantes presentes en los alimentos de sus hijos.<br>Retrotraducción 2: Soy capaz de realizar educación sanitaria a padres/ madres sobre los principales contaminantes presentes en los alimentos de sus hijos.<br><br>**Ítem final:** Sou capaz de realizar educação para a saúde aos pais sobre os principais contaminantes presentes na alimentação dos seus filhos.
**6.** NO soy capaz de identificar los riesgos medioambientales existentes en parques infantiles.<br><br>Traducción 1: Não sou capaz de identificar os riscos do meio ambiente existentes nos parques infantis.<br>Traducción 2: NÃO sou capaz de identificar os riscos ambientais existentes nos parques infantis.<br>Retrotraducción 1: NO soy capaz identificar los riesgos medioambientales existentes en parques infantiles.<br>Retrotraducción 2: NO soy capaz identificar los riesgos ambientales existentes en parques infantiles.<br><br>**Ítem final:** NÃO sou capaz de identificar os riscos ambientais existentes nos parques infantis.
**7.** Soy capaz de realizar educación sanitaria a padres y madres sobre las actuaciones para minimizar los riesgos medioambientales a los que está expuesto un niño o una niña cuando juega al aire libre.<br><br>Traducción 1: Sou capaz de realizar educação para a saúde aos pais sobre ações para minimizar os riscos do meio ambiente que uma criança está exposta ao brincar ao ar livre.

Traducción 2: Sou capaz de fazer educação para a saúde aos pais sobre ações para minimizar os riscos ambientais a que uma criança está exposta ao brincar ao ar livre.<br>Retrotraducción 1: Soy capaz de realizar educación sanitaria a padres/madres sobre las actuaciones para minimizar los riesgos ambientales a los que está expuesto un niño cuando juega al aire libre.<br>Retrotraducción 2: Soy capaz de realizar educación sanitaria a padres/madres sobre las actuaciones para minimizar los riesgos ambientales a los que está expuesto un niño cuando juega al aire libre.<br><br>**Ítem final:** Sou capaz de realizar educação para a saúde aos pais sobre ações para minimizar os riscos ambientais que uma criança está exposta ao brincar ao ar livre.
**8.** NO soy capaz de identificar los riesgos medioambientales existentes en la casa de un niño o una niña.<br><br>Traducción 1: Não consigo identificar os riscos do meio ambiente existentes em casa de uma criança.<br>Traducción 2: NÃO consigo identificar os riscos ambientais existentes em casa de uma criança.<br>Retrotraducción 1: NO soy capaz de identificar los riesgos medioambientales existentes en la casa de un niño.<br>Retrotraducción 2: NO soy capaz de identificar los riesgos ambientales existentes en la casa de un niño.<br><br>**Ítem final:** NÃO consigo identificar os riscos ambientais existentes em casa de uma criança.
**9.** Soy capaz de realizar educación sanitaria a padres y madres sobre los riesgos medioambientales existentes en el hogar.<br><br>Traducción 1: Sou capaz de realizar educação para a saúde aos pais sobre os riscos do meio ambiente existentes em casa.<br>Traducción 2: Sou capaz de fazer educação para a saúde aos pais sobre os riscos ambientais existentes em casa.<br>Retrotraducción 1: Soy capaz de realizar educación sanitaria a padres/madres sobre los riesgos ambientales existentes en casa.<br>Retrotraducción 2: Soy capaz de realizar educación sanitaria a padres/madres sobre los riesgos ambientales existentes en casa.<br><br>**Ítem final:** Sou capaz de realizar educação para a saúde aos pais sobre os riscos ambientais existentes em casa.
**10.** Soy capaz de identificar los riesgos medioambientales existentes en el colegio de un niño o una niña.<br><br>Traducción 1: Sou capaz de identificar os riscos do meio ambiente existentes na escola de uma criança.<br>Traducción 2: Sou capaz de identificar os riscos ambientais existentes na escola de uma criança.<br>Retrotraducción 1: Soy capaz de identificar los riesgos medioambientales existentes en el colegio de un niño.<br>Retrotraducción 2: Soy capaz de identificar los riesgos ambientales existentes en el colegio de un niño.

**Ítem final:** Sou capaz de identificar os riscos ambientais existentes na escola de uma criança.
**11.** NO soy capaz de identificar las actuaciones necesarias para combatir los riesgos medioambientales existentes en el colegio de un niño o una niña.<br><br>Traducción 1: Não consigo identificar as ações necessárias para combater os riscos do meio ambiente existentes na escola de uma criança.<br>Traducción 2: NÃO consigo identificar as ações necessárias para combater os riscos ambientais existentes na escola de uma criança.<br>Retrotraducción 1: NO soy capaz de identificar las actuaciones necesarias para combatir los riesgos ambientales existentes en el colegio de un niño.<br>Retrotraducción 2: NO soy capaz de identificar las actuaciones necesarias para combatir los riesgos ambientales existentes en el colegio de un niño.<br><br>**Ítem final:** NÃO consigo identificar as ações necessárias para combater os riscos ambientais existentes na escola de uma criança.
**12.** NO me siento capaz de desarrollar mi trabajo como enfermera en una Unidad de Salud Medioambiental Pediátrica.<br><br>Traducción 1: Não me sinto capaz de desenvolvero meu trabalho como enfermeiro numa Unidade de Saúde Ambiental Pediátrica – Unidade de Saúde Pública na área da Saúde Ambiental e Saúde Escolar.<br>Traducción 2: NÃO me sinto capaz de realizar o meu trabalho como enfermeiro numa Unidade de Saúde Ambiental Pediátrica – Unidade de Saúde Pública na área da Saúde Ambiental e Saúde Escolar (adaptação cultural).<br><br>Retrotraducción 1: NO me siento capaz de desarrollar mi trabajo como enfermera en una Unidad de Salud Pública en el área de Salud Ambiental Pediátrica y Salud Escolar (adaptación cultural).<br>Retrotraducción 2: NO me siento capaz de desarrollar mi trabajo como enfermera en una Unidad de Salud Ambiental Pediátrica y Salud Escolar (adaptación cultural).<br><br>**Ítem final:** NÃO me sinto capaz de realizar o meu trabalho como enfermeiro numa Unidade de Saúde Ambiental Pediátrica – Unidade de Saúde Pública na área da Saúde Ambiental e Saúde Escolar.

Respuestas: Totalmente en desacuerdo 1 2 3 4 5 Totalmente de acuerdo

TRADUCCIÓN: Totalmente em desacordo 1 2 3 4 5 Totalmente de acordo

COMENTARIOS:
