# Supplementary material for: Transcultural Adaptation of Environmental Health Questionnaire with Attitude, Knowledge, and Skills Scales for Portuguese Nursing Students
Source: Nurs Rep. 2025 Jan 8;15(1):13. doi: 10.3390/nursrep15010013 (PMC11767602; doi:10.3390/nursrep15010013)
Supplement: Supplementary file 1 [file nursrep-15-00013-s001.zip › Supplementary Materials C.pdf]

Supplementary Materials C: Inter-item correlations on the attitude scale (SANS_2)

Item	1	2	3	4	5
1	—				
2	0.688	—			
3	0.629	0.680	—		
4	0.629	0.794	0.726	—	
5	0.283	0.176	0.195	0.175	—
