# Supplementary material for: Transcultural Adaptation of Environmental Health Questionnaire with Attitude, Knowledge, and Skills Scales for Portuguese Nursing Students
Source: Nurs Rep. 2025 Jan 8;15(1):13. doi: 10.3390/nursrep15010013 (PMC11767602; doi:10.3390/nursrep15010013)
Supplement: Supplementary file 1 [file nursrep-15-00013-s001.zip › Supplementary Materials D.pdf]

## Supplementary Materials D: Yen's Q3 correlations on the knowledge scale (ChEHK-Q)

| | 1 | 2 | 3 | 4 | 5 | 6 | 7 | 8 | 9 | 10 | 11 | 12 | 13 | 14 | 15 | 16 | 17 | 18 | 19 | 20 | 21 | 22 | 23 | 24 | 25 | |
|---|---|---|---|---|---|---|---|---|---|---|---|---|---|---|---|---|---|---|---|---|---|---|---|---|---|---|
| 1 | 1,0000 | 0,0515 | 0,1475 | -0,1704 | 0,0390 | 0,1820 | -0,0073 | 0,0669 | -0,0755 | -0,0225 | -0,0581 | -0,0320 | -0,1128 | -0,0332 | -0,0789 | -0,1212 | -0,0898 | -0,0297 | -0,0019 | -0,0589 | -0,0597 | -0,0609 | -0,0202 | -0,0811 | -0,0864 | 0,0016 |
| 2 | 0,0515 | 1,0000 | -0,0305 | -0,0153 | -0,0299 | 0,0159 | -0,0146 | 0,0305 | 0,0176 | 0,0947 | -0,1055 | -0,1086 | -0,0110 | -0,0648 | -0,1654 | -0,1690 | -0,1194 | -0,1724 | -0,0014 | -0,0449 | 0,0871 | -0,0826 | 0,0018 | -0,1542 | -0,0426 | -0,0542 |
| 3 | 0,1475 | -0,0305 | 1,0000 | -0,0089 | -0,1051 | 0,1462 | -0,0387 | 0,1240 | -0,0180 | -0,1909 | 0,0552 | -0,0823 | -0,1317 | -0,0244 | -0,0859 | 0,0360 | -0,2519 | 0,0998 | -0,0381 | -0,1335 | -0,2212 | 0,0845 | -0,0787 | -0,0966 | -0,1905 | -0,1277 |
| 4 | -0,1704 | -0,0153 | -0,0089 | 1,0000 | -0,0938 | 0,0692 | 0,1530 | 0,0158 | -0,0592 | -0,0748 | -0,0689 | -0,0616 | -0,0023 | -0,0987 | -0,0986 | -0,0345 | -0,0339 | -0,0376 | -0,1387 | 0,0788 | 0,0082 | -0,0405 | -0,0053 | -0,0770 | -0,0220 | -0,0520 |
| 5 | 0,0390 | -0,0299 | -0,1051 | -0,0938 | 1,0000 | -0,0337 | -0,0868 | 0,0666 | -0,1339 | 0,0789 | 0,0119 | -0,0759 | -0,0175 | -0,0777 | 0,0311 | -0,0969 | -0,0105 | -0,0739 | 0,1384 | -0,0039 | 0,0519 | -0,1373 | -0,1369 | -0,1643 | 0,0629 | 0,1065 |
| 6 | 0,1820 | 0,0159 | 0,1462 | 0,0692 | -0,0337 | 1,0000 | 0,0686 | -0,0269 | 0,0077 | -0,1416 | -0,0916 | -0,0825 | -0,1986 | -0,0864 | 0,0044 | 0,0094 | -0,0879 | 0,0142 | -0,0534 | -0,1287 | -0,0921 | 0,0118 | -0,0493 | -0,0098 | -0,1520 | -0,0731 |
| 7 | -0,0073 | -0,0146 | -0,0387 | 0,1530 | -0,0868 | 0,0686 | 1,0000 | -0,0706 | -0,1408 | 0,0465 | -0,0781 | -0,0718 | -0,0451 | -0,0232 | -0,0816 | -0,0260 | -0,0036 | -0,1343 | -0,1070 | -0,0574 | -0,0890 | -0,0525 | -0,0430 | 0,0296 | -0,0182 | 0,0125 |
| 8 | 0,0669 | 0,0305 | 0,1240 | 0,0158 | 0,0666 | -0,0269 | -0,0706 | 1,0000 | 0,1950 | -0,1597 | -0,0441 | 0,0520 | -0,0521 | 0,0966 | -0,0689 | -0,0997 | -0,1865 | -0,0014 | -0,0257 | -0,2327 | -0,1117 | 0,0448 | -0,1835 | -0,1949 | -0,1630 | -0,1119 |
| 9 | -0,0755 | 0,0176 | -0,0180 | -0,0592 | -0,1339 | 0,0077 | -0,1408 | 0,1950 | 1,0000 | -0,1457 | 0,0094 | 0,1084 | -0,0759 | 0,0969 | 0,1039 | -0,0323 | -0,1705 | 0,0771 | -0,1704 | -0,1869 | -0,1158 | 0,0414 | -0,0944 | -0,1392 | -0,1617 | -0,0952 |
| 10 | -0,0225 | 0,0947 | -0,1909 | -0,0748 | 0,0789 | -0,1416 | 0,0465 | -0,1597 | -0,1457 | 1,0000 | -0,1156 | -0,0624 | 0,1322 | -0,1458 | -0,0287 | -0,1961 | 0,1007 | -0,2897 | 0,0232 | 0,0260 | -0,0255 | -0,1395 | 0,0204 | 0,0133 | 0,2023 | 0,0428 |
| 11 | -0,0581 | -0,1055 | 0,0552 | -0,0689 | 0,0119 | -0,0916 | -0,0781 | -0,0441 | 0,0094 | -0,1156 | 1,0000 | -0,0604 | -0,2123 | -0,0201 | 0,0132 | 0,0348 | -0,1844 | 0,0050 | 0,0135 | -0,0533 | -0,0368 | 0,0803 | -0,0480 | -0,1204 | -0,0453 | -0,0589 |
| 12 | -0,0320 | -0,1086 | -0,0823 | -0,0616 | -0,0759 | -0,0825 | -0,0718 | 0,0520 | 0,1084 | -0,0624 | -0,0604 | 1,0000 | 0,0916 | 0,0428 | 0,0867 | -0,0131 | -0,0911 | 0,1179 | -0,0793 | -0,1170 | 0,0055 | 0,0381 | -0,1717 | -0,0961 | -0,1346 | -0,2314 |
| 13 | -0,1128 | -0,0110 | -0,1317 | -0,0023 | -0,0175 | -0,1986 | -0,0451 | -0,0521 | -0,0759 | 0,1322 | -0,2123 | 0,0916 | 1,0000 | -0,0426 | -0,0609 | -0,0874 | 0,0930 | -0,1224 | 0,0335 | 0,1088 | 0,0623 | -0,1626 | -0,1175 | 0,0119 | -0,0489 | -0,0330 |
| 14 | -0,0332 | -0,0648 | -0,0244 | -0,0987 | -0,0777 | -0,0864 | -0,0232 | 0,0966 | 0,0969 | -0,1458 | -0,0201 | 0,0428 | -0,0426 | 1,0000 | -0,0989 | 0,0520 | 0,0158 | 0,0800 | -0,2044 | -0,1685 | -0,0218 | 0,0631 | -0,0760 | -0,1519 | -0,1386 | -0,1037 |
| 15 | -0,0789 | -0,1654 | -0,0859 | -0,0986 | 0,0311 | 0,0044 | -0,0816 | -0,0689 | 0,1039 | -0,0287 | 0,0132 | 0,0867 | -0,0609 | -0,0989 | 1,0000 | 0,1193 | -0,0543 | 0,0567 | -0,0581 | -0,0802 | -0,0614 | -0,0028 | -0,2371 | -0,0085 | -0,0759 | -0,0798 |
| 16 | -0,1212 | -0,1690 | 0,0360 | -0,0345 | -0,0969 | 0,0094 | -0,0260 | -0,0997 | -0,0323 | -0,1961 | 0,0348 | -0,0131 | -0,0874 | 0,0520 | 0,1193 | 1,0000 | 0,0753 | 0,0039 | -0,1018 | -0,1095 | -0,1689 | 0,0608 | -0,0112 | -0,0654 | -0,0595 | -0,1141 |
| 17 | -0,0898 | -0,1194 | -0,2519 | -0,0339 | -0,0105 | -0,0879 | -0,0036 | -0,1865 | -0,1705 | 0,1007 | -0,1844 | -0,0911 | 0,0930 | 0,0158 | -0,0543 | 0,0753 | 1,0000 | -0,1942 | -0,0171 | -0,0386 | -0,0281 | -0,0555 | 0,2013 | 0,0564 | 0,1411 | -0,0019 |
| 18 | -0,0297 | -0,1724 | 0,0998 | -0,0376 | -0,0739 | 0,0142 | -0,1343 | -0,0014 | 0,0771 | -0,2897 | 0,0050 | 0,1179 | -0,1224 | 0,0800 | 0,0567 | 0,0039 | -0,1942 | 1,0000 | -0,0094 | 0,0047 | -0,0263 | 0,1264 | -0,1640 | -0,1185 | -0,2126 | -0,1236 |
| 19 | -0,0019 | -0,0014 | -0,0381 | -0,1387 | 0,1384 | -0,0534 | -0,1070 | -0,0257 | -0,1704 | 0,0232 | 0,0135 | -0,0793 | 0,0335 | -0,2044 | -0,0581 | -0,1018 | -0,0171 | -0,0094 | 1,0000 | -0,0050 | -0,0335 | -0,1789 | -0,0771 | 0,0158 | 0,0372 | 0,0743 |
| 20 | -0,0589 | -0,0449 | -0,1335 | 0,0788 | -0,0039 | -0,1287 | -0,0574 | -0,2327 | -0,1869 | 0,0260 | -0,0533 | -0,1170 | 0,1088 | -0,1685 | -0,0802 | -0,1095 | -0,0386 | 0,0047 | -0,0050 | 1,0000 | 0,2363 | -0,1645 | 0,0027 | 0,1154 | 0,0435 | -0,0097 |
| 21 | -0,0597 | 0,0871 | -0,2212 | 0,0082 | 0,0519 | -0,0921 | -0,0890 | -0,1117 | -0,1158 | -0,0255 | -0,0368 | 0,0055 | 0,0623 | -0,0218 | -0,0614 | -0,1689 | -0,0281 | -0,0263 | -0,0335 | 0,2363 | 1,0000 | -0,2024 | -0,0220 | 0,0035 | 0,0105 | -0,0568 |
| 22 | -0,0609 | -0,0826 | 0,0845 | -0,0405 | -0,1373 | 0,0118 | -0,0525 | 0,0448 | 0,0414 | -0,1395 | 0,0803 | 0,0381 | -0,1626 | 0,0631 | -0,0028 | 0,0608 | -0,0555 | 0,1264 | -0,1789 | -0,1645 | -0,2024 | 1,0000 | 0,0099 | -0,0746 | -0,2368 | -0,1368 |
| 23 | -0,0202 | 0,0018 | -0,0787 | -0,0053 | -0,1369 | -0,0493 | -0,0430 | -0,1835 | -0,0944 | 0,0204 | -0,0480 | -0,1717 | -0,1175 | -0,0760 | -0,2371 | -0,0112 | 0,2013 | -0,1640 | -0,0771 | 0,0027 | -0,0220 | 0,0099 | 1,0000 | 0,1558 | 0,1587 | -0,0134 |
| 24 | -0,0811 | -0,1542 | -0,0966 | -0,0770 | -0,1643 | -0,0098 | 0,0296 | -0,1949 | -0,1392 | 0,0133 | -0,1204 | -0,0961 | 0,0119 | -0,1519 | -0,0085 | -0,0654 | 0,0564 | -0,1185 | 0,0158 | 0,1154 | 0,0035 | -0,0746 | 0,1558 | 1,0000 | 0,1514 | 0,1399 |
| 25 | -0,0864 | -0,0426 | -0,1905 | -0,0220 | 0,0629 | -0,1520 | -0,0182 | -0,1630 | -0,1617 | 0,2023 | -0,0453 | -0,1346 | -0,0489 | -0,1386 | -0,0759 | -0,0595 | 0,1411 | -0,2126 | 0,0372 | 0,0435 | 0,0105 | -0,2368 | 0,1587 | 0,1514 | 1,0000 | 0,1028 |
| 26 | 0,0016 | -0,0542 | -0,1277 | -0,0520 | 0,1065 | -0,0731 | 0,0125 | -0,1119 | -0,0952 | 0,0428 | -0,0589 | -0,2314 | -0,0330 | -0,1037 | -0,0798 | -0,1141 | -0,0019 | -0,1236 | 0,0743 | -0,0097 | -0,0568 | -0,1368 | -0,0134 | 0,1399 | 0,1028 | 1,0000 |
