# Supplementary material for: Transcultural Adaptation of Environmental Health Questionnaire with Attitude, Knowledge, and Skills Scales for Portuguese Nursing Students
Source: Nurs Rep. 2025 Jan 8;15(1):13. doi: 10.3390/nursrep15010013 (PMC11767602; doi:10.3390/nursrep15010013)
Supplement: Supplementary file 1 [file nursrep-15-00013-s001.zip › Supplementary Materials E.pdf]

Supplementary Materials E: Yen's Q3 correlations on the skill scale (ChEHS-Q)

	1	2	3	4	5	6	7	8	9	10	11	12
1	1,0000	-0,1610	0,1238	-0,2470	0,0405	-0,2726	0,0735	-0,2408	-0,0036	0,0909	-0,1976	-0,1931
2	-0,1610	1,0000	-0,2879	0,0097	-0,2374	0,0392	-0,3069	0,0490	-0,1984	-0,2862	0,1181	0,0794
3	0,1238	-0,2879	1,0000	0,1380	-0,0273	-0,4230	0,0196	-0,3034	-0,0628	0,1765	-0,3005	-0,1987
4	-0,2470	0,0097	0,1380	1,0000	-0,1065	-0,0120	-0,1588	-0,1508	-0,1894	-0,2653	-0,0916	-0,0390
5	0,0405	-0,2374	-0,0273	-0,1065	1,0000	-0,2799	0,2544	-0,3227	0,2416	0,0070	-0,2127	-0,2795
6	-0,2726	0,0392	-0,4230	-0,0120	-0,2799	1,0000	-0,0768	0,3207	-0,2480	-0,2171	0,1594	0,0779
7	0,0735	-0,3069	0,0196	-0,1588	0,2544	-0,0768	1,0000	-0,2921	0,1949	0,0922	-0,3473	-0,3065
8	-0,2408	0,0490	-0,3034	-0,1508	-0,3227	0,3207	-0,2921	1,0000	-0,2238	-0,1753	0,2016	0,1542
9	-0,0036	-0,1984	-0,0628	-0,1894	0,2416	-0,2480	0,1949	-0,2238	1,0000	0,1242	-0,1728	-0,2720
10	0,0909	-0,2862	0,1765	-0,2653	0,0070	-0,2171	0,0922	-0,1753	0,1242	1,0000	-0,1701	-0,2700
11	-0,1976	0,1181	-0,3005	-0,0916	-0,2127	0,1594	-0,3473	0,2016	-0,1728	-0,1701	1,0000	0,1074
12	-0,1931	0,0794	-0,1987	-0,0390	-0,2795	0,0779	-0,3065	0,1542	-0,2720	-0,2700	0,1074	1,0000
